# Landscape Genetics of *Schistocephalus solidus* Parasites in Threespine Stickleback (*Gasterosteus aculeatus*) from Alaska

**DOI:** 10.1371/journal.pone.0122307

**Published:** 2015-04-13

**Authors:** C. Grace Sprehn, Michael J. Blum, Thomas P. Quinn, David C. Heins

**Affiliations:** 1 Department of Ecology and Evolutionary Biology, Tulane University, New Orleans, LA, 70118, United States of America; 2 Tulane-Xavier Center for Bioenvironmental Research, Tulane University, New Orleans, LA, 70118, United States of America; 3 School of Aquatic and Fishery Sciences, University of Washington, Seattle, WA, 98195, United States of America; Bournemouth University, UNITED KINGDOM

## Abstract

The nature of gene flow in parasites with complex life cycles is poorly understood, particularly when intermediate and definitive hosts have contrasting movement potential. We examined whether the fine-scale population genetic structure of the diphyllobothriidean cestode *Schistocephalus solidus* reflects the habits of intermediate threespine stickleback hosts or those of its definitive hosts, semi-aquatic piscivorous birds, to better understand complex host-parasite interactions. Seventeen lakes in the Cook Inlet region of south-central Alaska were sampled, including ten in the Matanuska-Susitna Valley, five on the Kenai Peninsula, and two in the Bristol Bay drainage. We analyzed sequence variation across a 759 bp region of the mitochondrial DNA (mtDNA) cytochrome oxidase I region for 1,026 *S*. *solidus * individuals sampled from 2009-2012. We also analyzed allelic variation at 8 microsatellite loci for 1,243 individuals. Analysis of mtDNA haplotype and microsatellite genotype variation recovered evidence of significant population genetic structure within *S*. *solidus*. Host, location, and year were factors in structuring observed genetic variation. Pairwise measures revealed significant differentiation among lakes, including a pattern of isolation-by-distance. Bayesian analysis identified three distinct genotypic clusters in the study region, little admixture within hosts and lakes, and a shift in genotype frequencies over time. Evidence of fine-scale population structure in *S*. *solidus* indicates that movement of its vagile, definitive avian hosts has less influence on gene flow than expected based solely on movement potential. Observed patterns of genetic variation may reflect genetic drift, behaviors of definitive hosts that constrain dispersal, life history of intermediate hosts, and adaptive specificity of *S*. *solidus *to intermediate host genotype.

## Introduction

Gene flow can be a key determinant of evolutionary potential, particularly for organisms engaging in interactions shaped by adaptation (i.e., Red Queen dynamics; [1–7]. Evolutionary potential can be influenced, for example, by dispersal promoting the influx of new alleles affecting interactions between a highly specialized parasite and its host [5]. Similarly, barriers to gene flow can influence the nature of host-parasite interactions by impeding the influx of adaptive or maladaptive alleles. Adaptive interactions may reciprocally affect gene flow, which in turn can give rise to dynamic changes in evolutionary potential. Accordingly, characterizing gene flow in parasites, which is often understudied relative to their hosts, can help identify factors shaping adaptive interactions.

The genetic structure and connectivity of parasite populations can be strongly influenced by host dispersal, particularly when parasites lack free-living stages or exhibit strong host specificity [8–11]. Pocket gopher chewing lice (*Geomydooecus actuosi*) and pocket gopher hosts (*Thomomys bottae*) show similarly high levels of genetic differentiation, as gene flow and dispersal of *G*. *actuosi* is constrained by infrequent host-host contact [10,12]. Although gene flow in *Heligmosmoides polygyrus*, a nematode infecting field mice (*Apodemus sylvaticusi*), reflects the genetic structure of its host, the nematode exhibits higher levels of genetic diversity and differentiation [13]. In contrast, evidence of little to no population genetic structure in *Amblyomma americanum*, a widespread tick, has been attributed to low host specificity and high capacity for dispersal in its mammalian host [14,15].

It is less clear how connectivity among parasite populations corresponds to host dispersal potential when parasites have complex life cycles involving multiple hosts that differ in movement potential [6,16–19]. Few studies have examined how host movement shapes the population genetic structure of parasites with complex life cycles [4,11,20–22]. The most mobile host is thought to have the greatest influence over parasite population genetic structure, but attributing connectivity among parasite populations to the movement potential of one host or another can be difficult because asymmetries can arise due to a number of factors that are not necessarily related to dispersal, such as life history variation, that may also influence infection and transmission [10].

Here we evaluate spatial and temporal patterns of mitochondrial and multilocus nuclear genetic variation among populations of the parasitic tapeworm *Schistocephalus solidus* in the threespine stickleback (*Gasterosteus aculeatus*). The *Schistocephalus*-stickleback model system affords opportunities for examining relationships between host dispersal and population connectivity in parasites with complex life cycles because the threespine stickleback—which serves as the second intermediate host of *S*. *solidus*—has lower dispersal potential than the piscivorous birds that serve as definitive hosts [16,23,24]. We tested the hypothesis that *S*. *solidus* populations exhibit little to no genetic structure, as prior studies suggest that parasites cycling through both freshwater and more vagile terrestrial hosts tend to have less structured populations with higher gene flow than parasites with life cycles restricted to freshwater hosts [vagile host hypothesis; 10,11,20]. Prior work has shown, however, that *S*. *solidus* exhibits signatures of intraspecific genetic structure and host specificity in intermediate stickleback hosts, suggesting that the movement potential of definitive hosts may not define the genetic structure of *S*. *solidus* or that the movement potential of definitive hosts is not fully realized [16,25,26]. Accordingly, we tested the alternative hypothesis that *S*. *solidus* exhibits pronounced population genetic structure, reflecting the habits of threespine stickleback. Understanding the fine-scale genetic structure of *S*. *solidus* populations will help clarify how host movement can influence the evolutionary potential of parasites, and further illustrate how gene flow can shape the geography of host-parasite interactions.

## Materials and Methods

### Study system

*Schistocephalus solidus* is a diphyllobothriidean cestode that exhibits a complex lifecycle involving cyclopoid copepods and threespine stickleback fish as first and second intermediate hosts, respectively, and piscivorous birds from any one of over 40 species as definitive hosts [16,24,27]. The parasite is transmitted to sticklebacks through consumption of infected copepods containing procercoid larvae [16]. Once established in host fish, the parasite transforms into a plerocercoid larvae in the coelom of the stickleback, wherein almost all of the parasite’s growth occurs [24,27]. Multiple infections are common, and the total mass of the parasites can equal or exceed the mass of the host fish [28,29]. Infected threespine sticklebacks are consumed by definitive hosts, in whose gut the parasites undergo sexual maturation and reproduction [16,24,30]. Reproduction is accomplished by either selfing or cross-fertilization, depending on the number of parasites infecting a definitive host [16,24]. Bird feces transmit eggs into water where they hatch into free-living coracidia larvae that are consumed by cyclopoid copepods [16,24,31].

Threespine sticklebacks likely have more constrained movement potential than definitive bird hosts. The stickleback hosts sampled in this study were all residents of freshwater lakes, which typically remain in the lake of origin throughout life, unless connecting freshwater drainages allow movement among proximate lakes [32]. Threespine sticklebacks exhibit territoriality and localized homing behaviors up to 200 m; significant genetic divergence also has been found between populations in neighboring lakes [32–35]. Though comparably little is known about landscape-level movement of definitive hosts, many (e.g., loons and grebes) migrate from Alaska to the Gulf of Mexico during the winter and exhibit little to no population genetic divergence [36–39]. Breeding territories of Common Loons (*Gavia immer*), which are dominant predators of threespine stickleback in southern Alaska, average around 70 ha and are frequently held by the same mating pair over several years [37,40–42]. Adult loons without territories and loons that are unsuccessful in breeding may move to establish new territories in nearby lakes [37,40,41].

### Specimen collection

**Fig 1:**
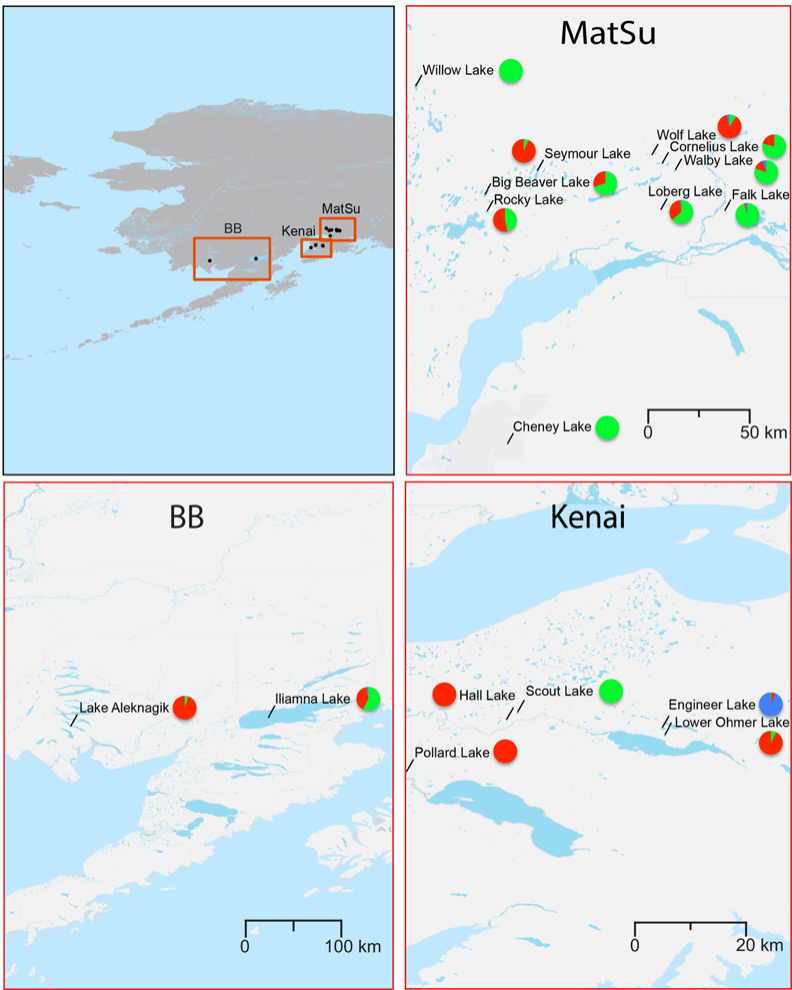
Map of Lakes. Overview of Alaska study site, including detail of the Matanuska-Sustina Valley, Kenai Peninsula, and Bristol Bay regions. Pie charts indicate the proportion of individuals of each genotype cluster by lake.

Infected threespine sticklebacks were sampled in late May to early June in 2009–2012 from 17 lakes in south-central and south-west Alaska. Collections were specifically approved by annual Fish Resource Permits from the Alaska Department of Fish and Game and animal care protocols from Tulane University Institutional Animal Care and Use Committee (protocols 0304R-UT-C and 0304R2) and the University of Washington Institutional Animal Care and Use Committee (protocol 3142–01). Fish were captured from ten lakes in the Matanuska-Susitna Valley (MatSu) north of Cook Inlet, five lakes on the Kenai Peninsula (Kenai) east of Cook Inlet, and two lakes west of Cook Inlet in Bristol Bay drainages (BB; Table 1; Fig 1). All of the sampled fish exhibited a benthic phenotype. Samples were taken with 3 mm and 6 mm wire-mesh minnow traps set near shore or with beach seines and tow nets. After euthanization with MS222, a ventral incision was made in each specimen prior to preservation in 95% ethanol. During necropsies all parasites were removed and preserved in 95% ethanol. Parasites were selected for analysis to maximize the number of hosts with large parasite loads (≥ 7 parasites) for within host and between lake comparisons (Table 1).

**Table 1:** Sampling and Diversity.

Lake Location		MtDNA Sampling								MtDNA Diversity					Microsatellite Sampling								Microsatellite Diversity				
Lake	Region	NP	NP(2009)	NP(2010)	NP(2011)	NP(2012)	NH	NH (>7/host)	NP (>7/host)	Total Haplotypes	Hap D	Pairwise D	Nucleotide D	K	NP	NP(2009)	NP(2010)	NP(2011)	NP(2012)	NH	NH (>7/host)	NP (>7/host)	Ho	He	N alleles	I	R
Big Beaver	MatSu	30	0	0	26	4	8	2	16	24	0.98	3.75	0.005	17.12	69	0	0	40	29	10	6	51	0.64	0.75	10.00	1.68	7.28
Cheney	MatSu	66	38	0	0	9	12	6	56	24	0.76	1.65	0.002	3.97	86	0	33	0	53	14	9	76	0.68	0.74	10.25	1.66	7.25
Cornelius	MatSu	47	0	10	0	56	29	0	0	30	0.96	4.84	0.006	17.12	56	43	0	0	13	36	1	6	0.71	0.77	11.50	1.81	8.60
Falk	MatSu	62	0	62	0	0	9	5	41	10	0.64	0.89	0.001	2.70	86	0	86	0	0	10	5	44	0.63	0.75	11.00	1.74	7.90
Loberg	MatSu	136	126	0	0	10	31	5	48	52	0.96	3.50	0.005	20.33	207	126	0	0	81	39	12	112	0.62	0.75	12.50	1.73	7.46
Rocky	MatSu	41	0	0	19	22	9	1	7	21	0.93	2.51	0.003	10.31	45	0	0	26	19	10	1	8	0.66	0.74	7.50	1.57	6.38
Seymour	MatSu	61	0	0	61	0	10	4	33	32	0.92	2.60	0.003	10.60	74	0	0	74	0	10	5	42	0.62	0.71	10.13	1.61	7.24
Walby	MatSu	309	219	0	45	45	41	19	239	105	0.91	2.66	0.004	11.12	290	232	0	27	31	40	13	196	0.64	0.74	14.75	1.76	7.91
Willow	MatSu	71	0	0	30	41	11	5	42	35	0.92	2.07	0.003	10.27	68	0	0	25	43	11	4	32	0.62	0.72	9.63	1.62	7.18
Wolf	Matsu	43	2	2	39	0	10	2	14	22	0.87	2.06	0.003	6.78	53	3	4	46	0	12	3	23	0.65	0.73	10.00	1.68	7.72
Engineer	Kenai	75	0	0	0	75	10	6	55	26	0.71	1.40	0.002	3.33	81	0	0	0	81	10	7	65	0.66	0.76	11.63	1.78	8.17
Hall	Kenai	2	0	0	0	2	2	0	0	2	1.00	3.00	0.004	2.00	1	0	0	0	1	1	0	0	1.00	1.00	2.00	0.20	-
Lower Ohmer	Kenai	28	0	0	0	28	21	0	0	17	0.92	2.23	0.003	8.53	26	0	0	0	26	20	0	0	0.60	0.72	8.13	1.59	7.33
Pollard	Kenai	3	0	0	0	3	3	0	0	1	0.00	0.00	0.000	1.00	4	0	0	0	4	4	0	0	0.56	0.66	3.50	1.04	-
Scout	Kenai	15	15	0	0	0	15	0	0	12	0.97	2.70	0.004	10.72	18	18	0	0	0	18	0	0	0.72	0.71	7.38	1.54	7.38
Aleknagik	BB	17	0	0	0	17	13	0	0	12	0.95	2.16	0.003	9.32	39	0	0	0	39	18	0	0	0.63	0.66	7.88	1.42	6.62
Iliamna	BB	20	0	0	0	20	6	2	14	9	0.71	1.31	0.002	3.03	40	0	0	0	40	11	2	14	0.62	0.74	9.38	1.66	7.69
Region																											
MatsSu	-	866	385	74	220	187	170	49	496	219	0.912	2.729	0.004	11.17	1034	404	123	238	269	192	59	590	0.64	0.76	19.13	1.80	7.49
Kenai	-	123	15	0	0	108	51	6	55	50	0.829	1.801	0.002	5.63	130	18	0	0	112	53	7	65	0.65	0.78	13.63	1.90	7.63
BB	-	37	0	0	0	37	19	2	14	18	0.850	1.709	0.002	5.78	79	0	0	0	79	29	2	14	0.62	0.71	10.63	1.62	7.16
Year																											
2009	-	400	-	-	-	-	102	13	190	122	0.95	3.24	0.004	20.15	422	-	-	-	-	109	13	204	0.65	0.73	15.75	1.71	4.82
2010	-	74	-	-	-	-	16	5	41	17	0.66	1.05	0.001	2.91	123	-	-	-	-	20	8	68	0.65	0.75	12.25	1.76	5.14
2011	-	220	-	-	-	-	36	16	129	81	0.89	2.32	0.003	8.99	238	-	-	-	-	36	16	127	0.63	0.76	13.75	1.78	5.05
2012	-	332	-	-	-	-	86	23	205	114	0.85	2.17	0.003	6.58	460	-	-	-	-	109	31	270	0.63	0.77	17.00	1.88	5.37
Total	-	1026	400	74	220	332	240	57	565	248	-	-	-	-	1034	404	123	238	269	274	59	590	-	-	-	-	-

NP: total number of parasites sequenced or genotyped; NP(2009–2012): number of parasites per year; NH: total number of hosts; NH(>7/host): number of hosts with greater than 7 parasites; NP(>7/host): number of parasites in hosts with greater than 7 parasites; Hap D: haplotype diversity in lake; Pairwise D: pairwise differences among haplotypes; Nucleotide D: nucleotide diversity among haplotypes; k: effective number of haplotypes; Ho: observed heterozygosity; He: expected heterozygosity; N alleles: average number of alleles over 8 loci; I: Shannon’s Information Index; R: average rarefied allelic richness across loci.

### Mitochondrial DNA sequence data collection and analysis

Genomic DNA from *S*. *solidus* specimens was extracted using the DNeasy Blood & Tissue extraction kit following the manufacturer’s standard protocol (Qiagen, Inc., Valencia, CA, USA). Polymerase chain reactions (PCRs) were performed to amplify 759 base pairs (bp) of the mitochondrial cytochrome oxidase 1 gene (*CO1*) region for 1,026 individuals (Table 1) using primers CYTW3F2 (CTAATTGGTGTGTGATCTGG TTTTG) and CYTW3R5 (GGAGTGGGAGCCCAACACAAG) [26]. PCRs were carried out using Mastercycler Pro thermocyclers (Eppendorf AG, Hamburg) with conditions following Nishimura et al. [26]. PCR products were purified using ExoSAP-It (USB, Affymetrix, Cleveland, OH). Cycle-sequencing reactions were then carried out using BigDye (Applied Biosystems, Foster City, CA), 3.2 mM primers, 4 μL ddH_2_O, and purified PCR product. Sequencing reactions were purified using Sephadex (GE Healthcare Biosciences, Pittsburgh, PA) according to manufacturer protocols. An ABI 3730xl DNA sequencer (Applied Biosystems, Foster City, CA) was used to electrophorese cycle-sequencing products. Sequencher v4.9 and Genalex v6.5 [43] software (Gene Codes Corp., Ann Arbor, MI) were used to align and edit the resulting DNA sequence data.

DNAsp v5.10.1 and Arlequin v3.5 were used to estimate the total number of haplotypes, haplotype diversity, pairwise differences among haplotypes, nucleotide diversity among haplotypes, and effective number of haplotypes for each lake, region, and year [44,45]. Effective number of haplotypes was calculated as the reciprocal of the sum of squared frequencies. The distribution of haplotype diversity and relationships among haplotypes were evaluated with Network by creating minimum spanning haplotype networks labeled according to lake, collection year, region, and genotype cluster assignment [46,47]. Using Arlequin v3.5 software [44], Analysis of Molecular Variance (AMOVA) was carried out to estimate the proportion of variance attributable to different hierarchical scales. AMOVAs based on 10,000 permutations were performed to assess patterns according to host and year, lake and year, lake and region, and genotype cluster. For AMOVAs examining variation among hosts, tests were limited to individuals from fish hosts with ≥ 7 parasites (totaling 565 individuals) to reduce possible bias from small sample sizes. Arlequin v3.5 [44] was also used to estimate global Φ_ST_ values and pairwise Φ_ST_ values between each lake and region.

To determine whether patterns of genetic differentiation reflect the movement potential or habits of hosts, pairwise routes of potential movement between all lakes were calculated using Google Earth. The most parsimonious pathways for travel were measured as straight line distances between lakes, as compared to river distances between lakes within and among watersheds. Pairwise Φ_ST_ values were compared to the between-lake linear and riverine distance matrices with Mantel’s tests to evaluate the strength of relationships and to detect signatures of isolation-by-distance. Mantel’s tests were performed in Genalex v6.5 with 10,000 permutations [43].

### Microsatellite data collection and analysis

A total of 1,243 individuals (Table 1) were genotyped at eight nuclear microsatellite loci—Scso33, Scso18, SsCAB6, Scso22, Scso29, Scso24, Scso35, and Scso9—following locus-specific amplification conditions described in Binz et al. [48]. Forward primers were labeled with HEX, FAM, or TAMRA fluorescent dyes to size amplicons against a 600 bp LIZ standard on an ABI 3730xl DNA sequencer. Electropherograms were scored and binned with GeneMarker v9.0 (Softgenetics, State College, PA).

Genetic diversity statistics, including expected (H_e_) and observed (H_O_) heterozygosity, average number of alleles (N alleles), and average rarefied allelic richness (R) were calculated for each lake, region, and year using Arlequin v3.5; Shannon’s Index (I) also was calculated using MSA software [44,49]. Hardy-Weinberg Equilibrium (HWE), linkage disequilibrium (LD), and F_IS_ values were calculated to test for equilibrium and to detect signatures of the Wahlund effect. HWE and F_IS_ values were calculated in Arlequin v3.5. Tests for LD were performed in Genepop v4.3, and p-values were compared following a Bonferroni correction (p < 0.0018) [50]. To examine the hierarchical distribution of microsatellite variation, Arlequin v3.5 was used to conduct AMOVAs with 10,000 permutations run per analysis [44]. Samples were grouped by host and year, host and lake, lake and region, and genotype cluster. For AMOVAs examining variation among hosts, tests were limited to individuals from fish with ≥7 parasites (totaling 565 individuals) to reduce possible bias from small sample sizes. Pairwise F_ST_ values were also calculated using Arlequin v3.5.

Structure v2.1 was used to assess patterns of genetic differentiation without *a priori* hypotheses of population structure [51]. We used a burn-in period of 10,000 iterations and 5 independent runs with 100,000 iterations where K (number of populations) was set iteratively from 1 to 17. Runs intended to evaluate the likelihood of structure corresponding to fish host were conducted with a data subset including parasites from hosts with ≥ 7 parasites where K was set from 1 and 6 iteratively. Analyses of structure among years and within clusters revealed in previous runs also were conducted where K was set from 1 to 4.

To determine whether patterns of genetic differentiation reflect the movement potential or habits of hosts, pairwise linearized F_ST_ values were compared to between-lake distance matrices (as described above) with Mantel’s tests to evaluate the strength of relationships and to detect signatures of isolation-by-distance. Mantel’s tests were performed in Genalex 6.5 with 10,000 permutations [43].

## Results

### MtDNA haplotype diversity and differentiation

Analysis of a 759 bp region of the mitochondrial *CO1* gene recovered 248 unique haplotypes in 1,026 individuals. Twelve polymorphisms separated the most divergent haplotypes from the most common haplotype (H1), which was found in all lakes except Hall Lake, and in all regions, years, and genotype clusters. Excluding Hall and Pollard lakes, which were not considered due to small sample sizes, the greatest haplotype diversity was detected in Big Beaver Lake (Table 1), where 24 unique haplotypes were recovered in 30 individuals. Minimum pairwise haplotype diversity occurred in Falk Lake, whereas maximum pairwise haplotype diversity occurred in Cornelius Lake (Table 1). Nucleotide diversity ranged from 0.0012 in Falk Lake to 0.0064 in Cornelius Lake, and the effective number of haplotypes ranged from 2.7 in Falk Lake to 20.32 in Loberg Lake. Haplotype diversity as well as pairwise and nucleotide diversity were consistent across regions (Table 1). The minimum effective number of haplotypes occurred in the Kenai region whereas the maximum was present in the MatSu region (Table 1). When partitioned by year, the highest haplotype diversity was recovered in 2009 and the lowest haplotype diversity was recovered in 2010. The effective number of haplotypes, and pairwise and nucleotide diversity were all highest in 2009 and lowest in 2010 (Table 1).

The haplotype networks revealed a high degree of heterogeneity across lakes and years (S1 Fig). There was a high degree of heterogeneity when haplotypes were identified by lake (S1 Fig), and though there was some evidence of structure by year, with early years (2009 and 2010) clustering relative to later years (2011 and 2012), some heterogeneity was still evident (S1 Fig).

Despite the absence of obvious structure in haplotype networks, mtDNA differentiation was significant across multiple hierarchical levels (Table 2). Significant global Φ_ST_ values were recovered, ranging from 0.027 to 0.08. The lowest global Φ_ST_ value was recovered when individuals were grouped by lake and region, whereas the highest global Φ_ST_ values were recovered when individuals were grouped by host and year (Table 2).

**Table 2:** Summary of AMOVA Results.

Fish host, Lake	Mitochondrial	Microsatellite
Percent variation among lakes	3.01	3.45
Percent variation among fish within lakes	4.62	2.80
Percent variation within fish	92.37	93.74
F_ST_	**0.08**	**0.06**
F_SC_	**0.05**	**0.02**
F_CT_	**0.03**	**0.03**
Fish host, Year		
Percent variation among years	2.63	2.61
Percent variation among fish within years	5.37	3.64
Percent variation within fish	92.00	93.75
F_ST_	**0.08**	**0.06**
F_SC_	**0.06**	**0.04**
F_CT_	**0.03**	**0.03**
Lake, Region		
Percent variation among regions	0.00	1.80
Percent variation among lakes within regions	3.45	2.29
Percent variation within lakes	96.83	95.91
F_ST_	**0.03**	**0.04**
F_SC_	**0.04**	**0.02**
F_CT_	0	**0.02**
Cluster		
Percent variation among clusters	1.75	10.31
Percent variation within clusters	98.25	89.69
F_ST_	**0.02**	**0.10**

Significant pairwise Φ_ST_ values by lake ranged from 0.0138 (Walby Lake and Cheney Lake) to 0.142 (Lower Ohmer Lake and Falk Lake; Table 3). Pairwise Φ_ST_ values by region were not significant except between MatSu and Kenai (0.0087; S1 Table). Mantel’s tests for associations between geographic distance and genetic distance (Φ_ST_ values) were not significant when distance was measured by stream length or straight line distances.

**Table 3:** Pairwise Φ_ST_ and F_ST_ Values by Lake.

	Cornelius	Loberg	Walby	Falk	Rocky	Big Beaver	Cheney	Wolf	Willow	Seymour	Iliamna	Aleknagik	Engineer	Hall	Pollard	Lower Ohmer	Scout
Cornelius	0	**.041**	**.05**	**.07**	**.084**	**.041**	**.097**	**.071**	**.096**	**.076**	**.026**	**.053**	**.107**	-.221	-.089	**.097**	.007
Loberg	**.005**	0	**.02**	**.047**	**.068**	**.034**	**.039**	**.036**	**.043**	**.04**	**.027**	**.034**	**.05**	-.135	-.114	**.074**	.017
Walby	.002	**.008**	0	**.015**	**.045**	**.032**	**.014**	.007	**.016**	**.018**	-.004	.015	**.014**	-.091	-.154	**.061**	.006
Falk	-.006	**.01**	-.001	0	**.1**	**.088**	**.05**	**.037**	**.068**	**.064**	.007	**.094**	**.046**	.263	-.116	**.142**	**.074**
Rocky	**.015**	**.008**	**.018**	**.023**	0	**.045**	**.07**	**.035**	**.049**	**.037**	**.042**	**.034**	**.056**	-.025	-.11	.003	**.062**
Big Beaver	-.002	.001	-.002	-.002	**.007**	0	**.057**	**.037**	**.039**	**.039**	.028	.027	**.063**	-.164	-.113	**.06**	.019
Cheney	-.002	**.003**	0	**.006**	.005	-.009	0	.007	.007	.009	**.032**	**.042**	**.017**	.096	-.165	**.083**	**.05**
Wolf	.001	**.002**	**.007**	**.006**	.009	0	-.001	0	.002	.004	.004	.009	.003	0	-.172	**.065**	**.034**
Willow	**.09**	**.073**	**.096**	**.1**	**.073**	**.076**	**.077**	**.074**	0	-.001	.022	.014	.007	-.015	-.146	**.07**	**.043**
Seymour	.001	.003	.001	0	.017	-.002	-.01	.007	.083	0	.02	.012	**.016**	-.068	-.148	**.059**	**.031**
Iliamna	**.068**	**.044**	**.078**	**.098**	**.028**	**.053**	**.056**	**.055**	**.042**	**.062**	0	.014	.01	.066	-.141	**.083**	.021
Aleknagik	**.077**	**.056**	**.079**	**.105**	**.032**	**.059**	**.063**	**.062**	**.062**	**.071**	.014	0	.023	-.058	-.128	**.072**	**.041**
Engineer	**.064**	**.043**	**.069**	**.081**	**.033**	**.051**	**.05**	**.05**	**.032**	**.054**	.001	.014	0	.115	-.164	**.088**	**.066**
Hall	.102	.12	.125	.14	.133	.107	.101	.131	.174	.095	.219	.262	.187	0	.25	.086	-.148
Pollard	.009	.024	.004	.005	**.053**	.012	-.002	.034	**.135**	.004	**.142**	**.135**	**.119**	.097	0	-.091	-.128
Lower Ohmer	**.011**	**.025**	**.013**	-.006	**.042**	**.024**	.007	**.014**	**.116**	**.024**	**.115**	**.113**	**.108**	.098	**.039**	0	**.09**
Scout	**.01**	**.018**	**.008**	-.002	**.042**	**.016**	-.003	**.023**	**.126**	.011	**.114**	**.13**	**.109**	.067	.006	**.025**	0

Φ_ST_ values are above the diagonal and F_ST_ values are below the diagonal. Significant values are bold (p>0.05).

### Microsatellite genetic diversity and differentiation

Microsatellite-based measures of genetic diversity were similar across lakes except for Hall Lake and Pollard Lake, which were outliers likely due to small sample sizes. Observed heterozygosity was consistent across all lakes (Table 1) with values ranging from 0.61 (Lower Ohmer Lake) to 0.72 (Scout Lake). Only a few instances of departures from HWE were detected in a subset of populations at select loci (S2 Table). Significant F_IS_ values were present in ten lakes, ranging from 0.04 (Falk Lake) to 0.167 (Lower Ohmer Lake; S2 Table). All loci were found to be in LD except in 4 of 476 comparisons between loci within lakes (Walby Lake, Scso18 & SsCAB6; Walby Lake, Scso18 & Scso35; Rocky Lake, Scso18 & Scso24; Iliamna Lake, Scso35 & Scso9). Specimens from Engineer Lake exhibited the highest average number of alleles, and Scout Lake exhibited the lowest (Table 1). Shannon Index values ranged from 1.42 (Aleknagik Lake) to 1.81 (Cornelius Lake). Rarefied allelic richness ranged from 6.38 (Rocky Lake) to 8.60 (Cornelius Lake). Individuals from the MatSu region exhibited the highest average number of alleles, and individuals from the BB region exhibited the lowest average number of alleles. Shannon Index by region ranged from 1.62 (BB) to 1.8 (MatSu). Rarefied allelic richness ranged from 7.16 (BB) to 7.63 (Kenai). When partitioned by year, the greatest Shannon Index values and average number of alleles was present in 2012 and the smallest respective values occurred in 2010 (Table 1).

Microsatellite-based estimates of genetic differentiation were highly significant (Table 2). All global F_ST_ values, which ranged from 0.04 to 0.062, were highly significant (Table 2). The lowest global F_ST_ value was recovered when individuals were grouped by host and lake, while the highest global F_ST_ value was recovered when individuals were grouped by host and year (Table 2). Pairwise F_ST_ values by lake ranged from 0.0018 to 0.142, with many comparisons being highly significant (Table 3). The highest pairwise values were between Pollard and Iliamna (0.142), Pollard and Aleknagik (0.135), and Scout and Aleknagik (0.13; Table 3). Pairwise F_ST_ values between regions were all significant (S1 Table). The highest pairwise value was between MatSu and Kenai (0.041), and the lowest was between BB and Kenai (0.022; S1 Table).

**Fig 2:**
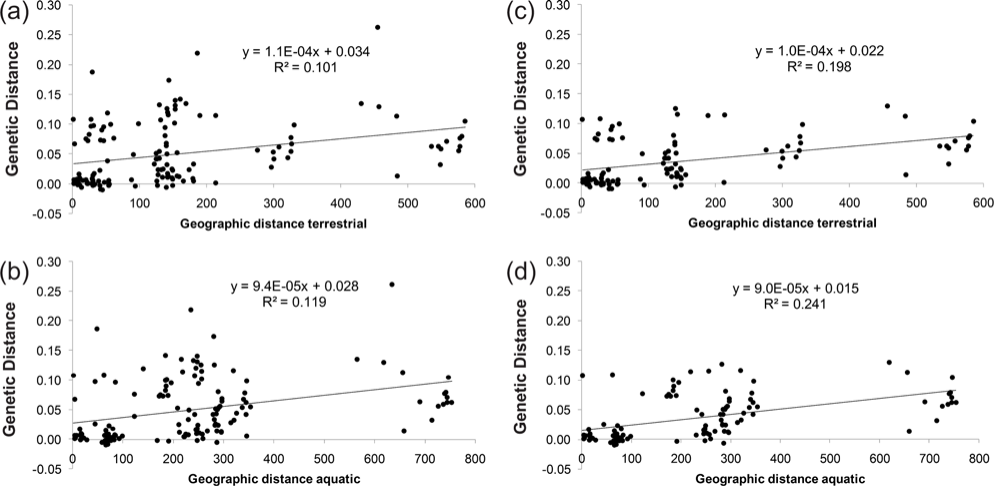
Mantel Tests. Microsatellite based estimates of genetic differentiation (linearized F_ST_) compared to (a) Euclidian distance between lakes, (b) Euclidian distance between lakes distance by streams without Hall and Pollard lakes to account for sample size variation, and (c) distance by streams, and (d) distance by streams without Hall and Pollard lakes.

Mantel’s tests revealed evidence of isolation by distance. When all sites were considered, slightly stronger non-significant trends were recovered when distance was measured via stream length (R_xy_ = 0.35, R^2^ = 0.12, P = 0.072) than by straight line distances (R_xy_ = 0.32, R^2^ = 0.10, P = 0.086). When Hall and Pollard lakes were removed from the calculation to control for small sample sizes, highly significant relationships were detected, with distance based on stream length (R_xy_ = 0.491, R^2^ = 0.24, P = 0.007) being a stronger predictor than straight line distances (R_xy_ = 0.445, R^2^ = 0.198, P = 0.021; Fig 2).

The results of the Bayesian clustering analysis in STRUCTURE indicated a peak in posterior probabilities (-ln P(D)) at K = 2, and ΔK values also detected the coarsest level of genetic structure at K = 2 (Fig 1, S2 Fig). Most individuals from Aleknagik, Engineer, Hall, Lower Ohmer, Pollard, Seymour, and Wolf lakes were assigned to one cluster, and most individuals in Cheney, Falk, Willow, and Scout lakes were assigned to a second cluster (Fig 1, S2 Fig). Individuals from Big Beaver, Cornelius, Iliamna, Loberg, Rocky, and Walby lakes were assigned to both clusters. Most individuals in a host were assigned completely to one cluster or the other; only six fish hosted *S*. *solidus* from more than one cluster, and only a few hosts carried individuals with admixed genotypes (S2 Fig). The frequency of the two clusters varied over time; the second cluster became more frequent in the latter two years of the study (S2 Fig). At K = 5, individuals from Engineer Lake and some individuals from Walby Lake were assigned to a third cluster independent of most other samples (Fig 1, S2 Fig). When individuals from the two primary genetic clusters were run separately as subsets (i.e., cluster one and cluster two respectively), individuals from cluster one were assigned to two genetic clusters, and individuals in cluster two tended towards incomplete assignment when run as a subset, as was observed when all individuals were run together (S2 Fig).

## Discussion

The evolutionary potential of a parasite can depend on gene flow, which frequently reflects host dispersal [52,53]. This study tested the hypotheses that the genetic structure of *S*. *solidus* reflects the habits of its intermediate host or, alternatively, those of its definitive hosts. We evaluated spatial and temporal patterns of genetic variation and found evidence of significant population genetic structure in *S*. *solidus* infecting threespine sticklebacks in lakes throughout south-central Alaska. The observed patterns of genetic differentiation and diversity showed that gene flow is constrained, and that variability over space and time likely reflects factors other than the movement of the most vagile host. We recovered significant mtDNA and microsatellite-based estimates of genetic differentiation, with genetic structure recovered in frequency-based and Bayesian clustering analysis (Tables 1 and 2; Figs 1 and 2). Even though levels of differentiation found in fish and parasites are frequently higher than in other systems, F_ST_ and Φ_ST_ values in *S*. *solidus* are comparably higher than levels of genetic differentiation in similar host-parasite systems [54–57]. For example, much lower levels of differentiation have been found in *Ligula intestinalis*, another geographically widespread cestode parasite, which has been attributed to transportation of eggs by vagile piscivorous birds [10,58].

Hierarchical signatures of genetic differentiation were attributable to differences among stickleback hosts within years and among lakes, indicating that parasite populations may be defined by intermediate host identity, host cohort, or lake of origin (Table 2). Though measures of hierarchical genetic differentiation likely captured other sources of differentiation due to samples being taken over time, pairwise measures of differentiation suggest that *S*. *solidus* is significantly differentiated among lakes (Table 3). Clustering analysis also suggests the presence of two populations across south-central Alaska that differ in frequency among lakes (Fig 1, S2 Fig). Lakes generally were assigned completely to one cluster or the other, though some (e.g. Big Beaver, Cornelius, Iliamna, Loberg, Rocky, and Walby) harbored both clusters (Fig 1). The highest numbers of effective mitochondrial haplotypes corresponded to lakes that harbored both clusters (Table 1). This indicates that the movement of its vagile, definitive hosts influences gene flow in *S*. *solidus* less than would be expected based on movement potential [4,9,10,20,59]. The observed patterns of genetic variation in *S*. *solidus* could be attributed to one or more possible mechanisms, including genetic drift, behaviors of definitive hosts constraining dispersal, feeding or movement of intermediate hosts, and adaptive specificity of *S*. *solidus* to intermediate host genotype.

### Genetic variation

The high levels of genetic diversity observed in *S*. *solidus* (Tables 1, 2, and 3; Figs 1 and 2) likely reflects transmission across a complex life cycle. High levels of genetic diversity in *S*. *solidus* may arise because a single stickleback host can contain multiple parasite genotypes, as copepods containing cestodes are consumed individually and in great numbers by fish. Definitive avian host individuals may in turn consume many stickleback hosts [60]. Similar levels of genetic diversity have been observed in other host-parasite systems where intermediate hosts harbor multiple parasite genotypes and definitive hosts consume several intermediate hosts [11,61].

The observed hierarchical population structure in *S*. *solidus* also is likely an outcome of a complex life cycle. Observed patterns of genetic differentiation suggest that parasites within a stickleback intermediate host can be considered a subpopulation comprised of discrete or overlapping generational cohorts, and lakes a collection of multiple subpopulations [10]. Though subpopulations are well defined within intermediate hosts, subsequent mixing can occur within a lake because multiple infected fish may be consumed by a single definitive host. Further geographic nesting could reflect definitive hosts feeding and shedding parasites within a particular lake or region [10]; if definitive hosts feed on intermediate hosts in a single lake, the subsequent generation of parasites may be returned to the same or nearby lake if shedding occurs soon after consumption of infected stickleback.

### Population connectivity

The observed patterns of genetic differentiation in *S*. *solidus* indicate that population connectivity is lower than what would be expected from the high dispersal potential of its definitive hosts. Semi-aquatic birds like Common Loons can readily carry *S*. *solidus* among lakes. Yet, 2.96% of mtDNA haplotype variation is attributable to differences among lakes (Table 2). Pairwise F_ST_ values based on microsatellite allelic variation also indicate that *S*. *solidus* in nearby lakes exhibit significant levels of genetic differentiation (e.g. Engineer and Lower Ohmer, F_ST_ = 0.11; Table 3). Additionally, we found that *S*. *solidus* exhibits signatures of isolation-by-distance, suggesting that there are stronger geographical barriers to gene flow in *S*. *solidus* than has been previously thought (Fig 2). The signature of isolation-by-distance was more pronounced when distance corresponded to riverine movement corridors, suggesting that watershed boundaries could be important in mediating gene flow among lakes. The movement of definitive avian hosts might be constrained if, for example, flight patterns correspond to drainage topography rather than straight-line pathways or if avian hosts exhibit site fidelity. It is also possible, however, that dispersal of definitive hosts does not coincide with periods of infection, which can be relatively brief (i.e., days to weeks). Reproduction and shedding of *S*. *solidus* also might occur prior to dispersal of definitive hosts. If so, then the observed pattern of IBD among lakes could be a reflection of small effective population sizes and genetic drift arising from stochastic synchronicity between infection, reproduction and dispersal of definitive hosts.

The strong signatures of temporal variation observed across the four-year study period appear to be tied to the habits of stickleback hosts. Among year differences in mtDNA haplotype frequencies (Table 2) and evidence of replacement of one by another microsatellite genotypic cluster over the study period (S2 Fig) are consistent with the feeding ecology of stickleback hosts [53,62]. Sticklebacks typically cease accumulating parasites when their diet shifts away from copepods late in their first year after hatching, which could result in a two year turnover cycle of genotypic clusters (i.e., generational cohorts) in lakes corresponding to the lifespan of stickleback hosts [16,23,32,63]. It is also possible, however, that transmission cycles could be acting in conjunction with genetic drift and constrained or variable definitive host movement to promote spatial and temporal genetic differentiation in *S*. *solidus*.

The observed patterns of restricted gene flow may also be an outcome of intermediate host specificity within *S*. *solidus*. Nishimura et al. (2011) recovered phylogenetic evidence indicating that continental patterns of genetic differentiation in *Schistocephalus* parasites reflect specificity between threespine and ninespine stickleback intermediate hosts [26,64]. Evidence of fine-scale geographical differences in parasite responses to host immune function suggests that *S*. *solidus* may also exhibit adaptive specificity to evolutionary lineages of threespine stickleback [65,66]. If so, then the observed patterns of genetic variation might reflect differential infection of the two genetically distinct but morphologically identical clades of threespine stickleback that occur in south-central Alaska [62,65,67,68]. Though the low percentage of hosts (~2.5%) infected by parasites assigned to two genotypic clusters and evidence of high genetic diversity and genetic differentiation in *S*. *solidus* are consistent with this scenario, further study is warranted to determine the likelihood of adaptive host specificity in mediating gene flow in *S*. *solidus* [3,55,65,69]. Comparisons of host haplotype to parasite genotype could, for example, reveal whether patterns of genetic relatedness are correlated. Additional comparisons focusing on functional genes associated with immune response could also prove informative, especially with reference to further information on life history and demographic factors that may influence host-parasite interactions.

## Supporting Information

S1 Fig Haplotype Networks. Minimum spanning networks were constructed in Network and colored by (a) lake, (b) year collected, and (c) genotypic cluster assignment (K = 3). Lines represent one base pair difference between haplotypes, and black nodes represent transitional mutations.
(TIFF)

S2 Fig Bayesian Cluster Analysis. Grouped by lake, with (a) K = 2, (b) K = 5, by (c) year collected K = 2, (d) fish host, with only hosts with greater than seven parasites, K = 2, (e) cluster one K = 2, (f) cluster two K = 2, and (g) mean ln P(D) plot.
(TIFF)

S1 Table Pairwise Φ_ST_ and F_ST_ Values by Region. Φ_ST_ values are above the diagonal and F_ST_ values are below the diagonal. Significant values are bold (p>0.05). MatSu = Matanuska-Susitna Valley; Kenai = Kenai Peninsula; BB = Bristol Bay region.
(DOCX)

S2 Table Estimates of Hardy-Weinberg Equilibrium. N: number of individuals per lake; H_E_: expected heterozygosity; H_O_: observed heterozygosity; F_IS_: inbreeding coefficient. Bolded H_O_ values indicate significant departures from Hardy-Weinberg Equilibrium (p<0.05).
(DOCX)
