# Supplementary figures and images for: Landscape Genetics of Schistocephalus solidus Parasites in Threespine Stickleback (Gasterosteus aculeatus) from Alaska
Source: PLoS One. 2015 Apr 13;10(4):e0122307. doi: 10.1371/journal.pone.0122307 (PMC4395347; doi:10.1371/journal.pone.0122307)

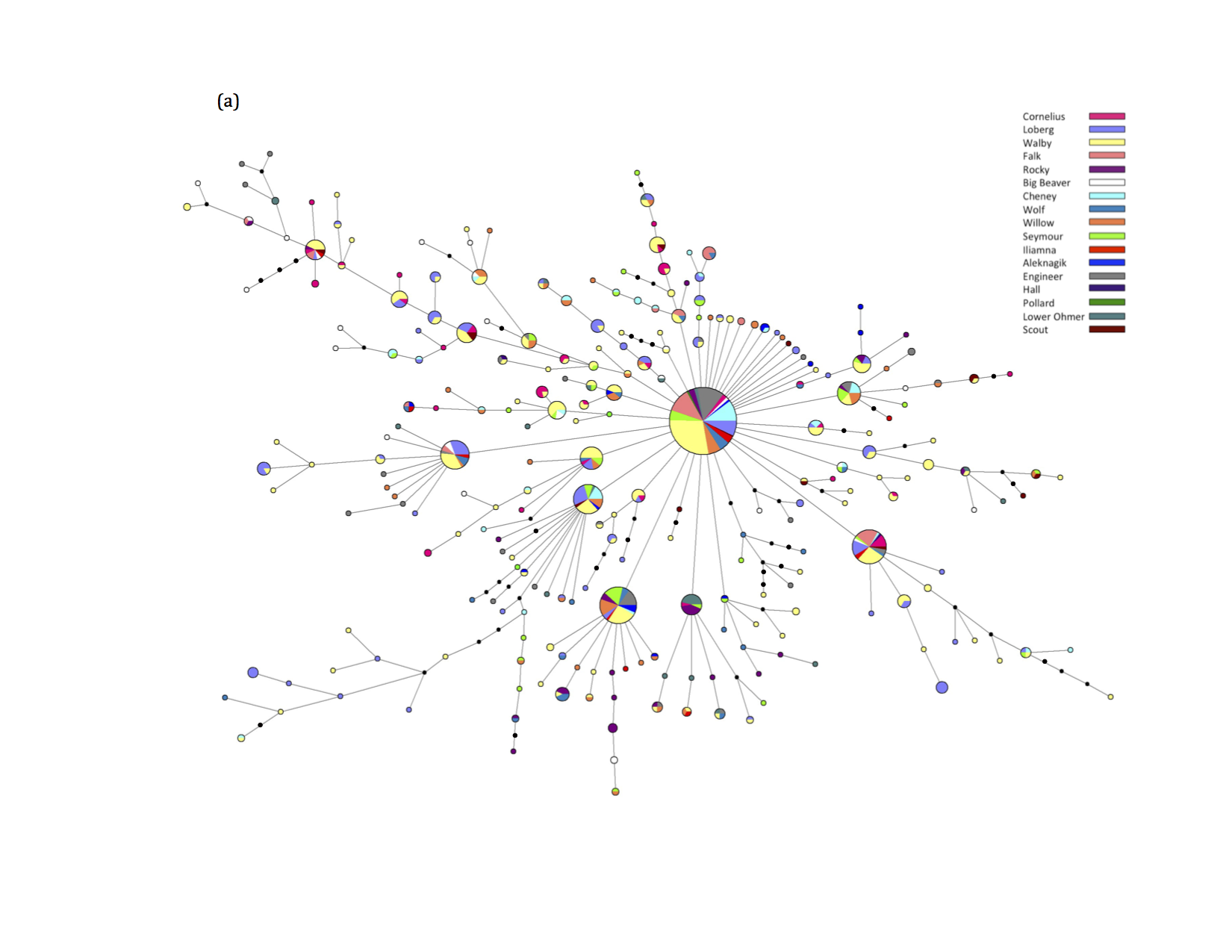

Supplement: S1 Fig — Minimum spanning networks were constructed in Network and colored by (a) lake, (b) year collected, and (c) genotypic cluster assignment (K = 3). Lines represent one base pair difference between haplotypes, and black nodes represent transitional mutations. (TIFF) [file pone.0122307.s001.tiff]

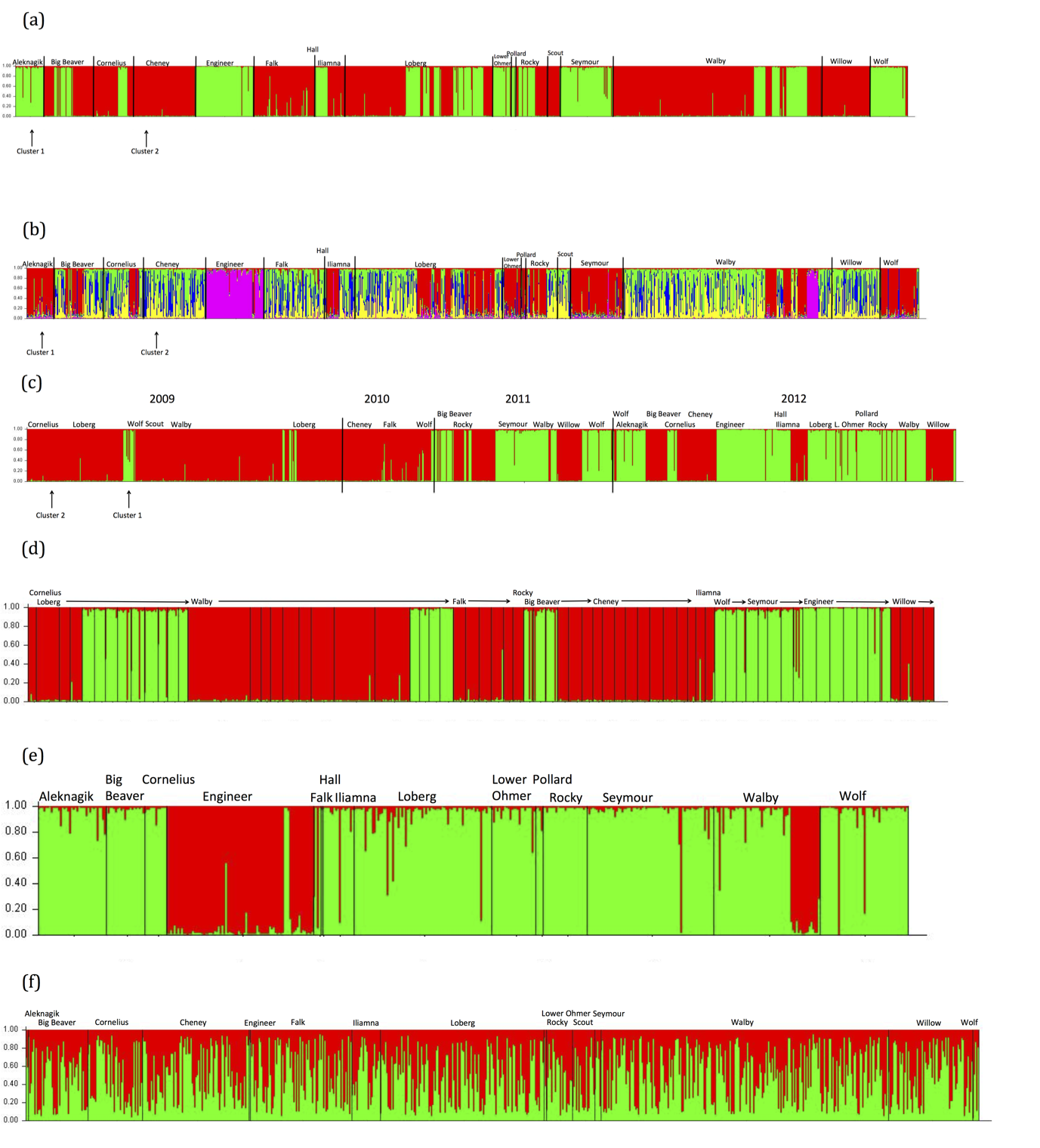

Supplement: S2 Fig — Grouped by lake, with (a) K = 2, (b) K = 5, by (c) year collected K = 2, (d) fish host, with only hosts with greater than seven parasites, K = 2, (e) cluster one K = 2, (f) cluster two K = 2, and (g) mean ln P(D) plot. (TIFF) [file pone.0122307.s002.tiff]
