# Supplementary material for: Landscape Genetics of Schistocephalus solidus Parasites in Threespine Stickleback (Gasterosteus aculeatus) from Alaska
Source: PLoS One. 2015 Apr 13;10(4):e0122307. doi: 10.1371/journal.pone.0122307 (PMC4395347; doi:10.1371/journal.pone.0122307)
Supplement: S1 Table — ΦST values are above the diagonal and FST values are below the diagonal. Significant values are bold (p>0.05). MatSu = Matanuska-Susitna Valley; Kenai = Kenai Peninsula; BB = Bristol Bay region. (DOCX) [file pone.0122307.s003.docx]

**S1 Table. Pairwise Φ_ST_ and F_ST_ Values by Region**.

|  | MatSu | BB | Kenai |
| --- | --- | --- | --- |
| MatSu | 0 | .006 | .009 |
| BB | **.026** | 0 | **.009** |
| Kenai | **.041** | **.022** | 0 |

Φ_ST_ ­values are above the diagonal and F_ST_ values are below the diagonal. Significant values are bold (p>0.05).
