# Supplementary material for: Landscape Genetics of Schistocephalus solidus Parasites in Threespine Stickleback (Gasterosteus aculeatus) from Alaska
Source: PLoS One. 2015 Apr 13;10(4):e0122307. doi: 10.1371/journal.pone.0122307 (PMC4395347; doi:10.1371/journal.pone.0122307)
Supplement: S2 Table — N: number of individuals per lake; HE: expected heterozygosity; HO: observed heterozygosity; FIS: inbreeding coefficient. Bolded HO values indicate significant departures from Hardy-Weinberg Equilibrium (p<0.05). (DOCX) [file pone.0122307.s004.docx]

| **S2 Table. Estimates of Hardy-Weinberg Equilibrium** | Population | Big Beaver | Cheney | Cornelius | Falk | Loberg | Rocky | Seymour | Walby | Willow | Wolf | Engineer | Hall | Lower Ohmer | Pollard | Scout | Aleknagik | Iliamna |
| --- | --- | --- | --- | --- | --- | --- | --- | --- | --- | --- | --- | --- | --- | --- | --- | --- | --- | --- |
| Locus | N | 30 | 66 | 47 | 62 | 136 | 41 | 61 | 309 | 71 | 43 | 75 | 2 | 28 | 3 | 15 | 17 | 20 |
| Scso33 | H_E_ | 0.74 | 0.80 | 0.79 | 0.82 | 0.76 | 0.70 | 0.72 | 0.81 | 0.75 | 0.82 | 0.77 | - | 0.84 | 0.86 | 0.80 | 0.73 | 0.80 |
|  | H_O_ | 0.69 | 0.82 | 0.75 | 0.73 | 0.75 | 0.73 | **0.68** | **0.77** | 0.70 | **0.79** | **0.67** | - | 0.77 | 1.00 | 0.78 | 0.81 | 0.81 |
|  | F_IS_ | 0.064 | -0.022 | 0.048 | 0.103 | 0.018 | -0.047 | 0.061 | 0.053 | 0.069 | 0.034 | 0.130 | - | 0.086 | -0.200 | 0.034 | -0.121 | -0.013 |
| Scso18 | H_E_ | 0.69 | 0.70 | 0.71 | 0.72 | 0.71 | 0.75 | 0.72 | 0.70 | 0.73 | 0.68 | 0.75 | - | 0.74 | 0.82 | 0.71 | 0.69 | 0.71 |
|  | H_O_ | 0.67 | 0.69 | 0.67 | **0.69** | 0.70 | 0.73 | **0.71** | **0.64** | 0.68 | 0.73 | 0.70 | - | 0.81 | 0.75 | 0.83 | 0.70 | 0.69 |
|  | F_IS_ | 0.025 | 0.008 | 0.050 | 0.031 | 0.009 | 0.017 | 0.014 | 0.080 | 0.071 | -0.078 | 0.057 | - | -0.088 | 0.100 | -0.178 | -0.012 | 0.021 |
| SsCAB6 | H_E_ | 0.84 | 0.83 | 0.89 | 0.85 | 0.82 | 0.79 | 0.79 | 0.79 | 0.82 | 0.84 | 0.86 | 1.00 | 0.84 | 0.43 | 0.80 | 0.82 | 0.88 |
|  | H_O_ | **0.41** | **0.53** | **0.63** | **0.44** | **0.52** | **0.53** | **0.53** | **0.55** | **0.50** | **0.44** | **0.51** | 1.00 | **0.38** | 0.00 | **0.78** | **0.46** | **0.50** |
|  | F_IS_ | 0.519 | 0.362 | 0.299 | 0.483 | 0.366 | 0.326 | 0.333 | 0.313 | 0.392 | 0.481 | 0.403 | 0.000 | 0.546 | 1.000 | 0.031 | 0.441 | 0.434 |
| Scso22 | H_E_ | 0.86 | 0.81 | 0.84 | 0.86 | 0.79 | 0.77 | 0.86 | 0.87 | 0.87 | 0.84 | 0.91 | - | 0.89 | 0.64 | 0.86 | 0.77 | 0.82 |
|  | H_O_ | 0.81 | **0.62** | 0.84 | **0.69** | **0.59** | 0.76 | **0.67** | **0.68** | **0.73** | 0.77 | **0.67** | - | **0.81** | 0.75 | 0.83 | 0.75 | 0.74 |
|  | Fis | 0.061 | 0.242 | 0.000 | 0.205 | 0.261 | 0.012 | 0.226 | 0.211 | 0.161 | 0.084 | 0.274 | - | 0.091 | -0.200 | 0.029 | 0.032 | 0.110 |
| Scso29 | H_E_ | 0.62 | 0.69 | 0.72 | 0.72 | 0.70 | 0.66 | 0.72 | 0.67 | 0.70 | 0.72 | 0.68 | - | 0.55 | 0.75 | 0.55 | 0.56 | 0.50 |
|  | H_O_ | 0.59 | 0.71 | 0.81 | **0.56** | **0.56** | 0.66 | 0.65 | **0.58** | 0.64 | **0.60** | 0.73 | - | 0.56 | 0.75 | 0.50 | 0.55 | 0.50 |
|  | F_IS_ | 0.044 | -0.016 | -0.133 | 0.224 | 0.198 | -0.006 | 0.101 | 0.129 | 0.092 | 0.170 | -0.067 | - | -0.018 | 0.000 | 0.089 | 0.008 | 0.001 |
| Scso24 | H_E_ | 0.83 | 0.82 | 0.82 | 0.79 | 0.83 | 0.86 | 0.83 | 0.80 | 0.81 | 0.85 | 0.83 | - | 0.83 | 0.75 | 0.85 | 0.71 | 0.79 |
|  | H_O_ | 0.78 | 0.87 | 0.81 | 0.80 | 0.82 | 0.87 | **0.69** | 0.76 | 0.75 | 0.77 | 0.79 | - | 0.73 | 0.50 | 0.83 | 0.69 | **0.66** |
|  | F_IS_ | 0.053 | -0.059 | 0.008 | -0.017 | 0.009 | -0.006 | 0.168 | 0.053 | 0.081 | 0.095 | 0.054 | - | 0.120 | 0.368 | 0.019 | 0.021 | 0.168 |
| Scso35 | H_E_ | 0.67 | 0.45 | 0.6 | 0.49 | 0.69 | 0.71 | 0.35 | 0.42 | 0.31 | 0.38 | 0.54 | - | 0.42 | 0.43 | 0.29 | 0.35 | 0.71 |
|  | H_O_ | **0.42** | 0.43 | **0.45** | 0.52 | **0.39** | **0.51** | **0.33** | **0.59** | 0.27 | **0.34** | **0.54** | - | **0.23** | 0 | 0.33 | 0.43 | **0.33** |
|  | F_IS_ | 0.378 | 0.034 | 0.250 | -0.056 | 0.438 | 0.285 | 0.063 | 0.294 | 0.121 | 0.117 | -0.014 | - | 0.459 | 1.000 | -0.140 | -0.224 | 0.536 |
| Scso9 | H_E_ | 0.73 | 0.78 | 0.76 | 0.76 | 0.70 | 0.71 | 0.70 | 0.71 | 0.75 | 0.73 | 0.74 | 1.00 | 0.68 | 0.61 | 0.80 | 0.63 | 0.71 |
|  | H_O_ | 0.70 | 0.76 | 0.73 | **0.62** | **0.62** | **0.47** | 0.73 | 0.69 | 0.68 | **0.75** | 0.71 | 1.00 | 0.54 | 0.75 | 0.83 | 0.61 | 0.72 |
|  | F_IS_ | 0.040 | 0.032 | 0.040 | 0.185 | 0.113 | 0.345 | -0.043 | 0.029 | 0.100 | -0.018 | 0.032 | 0.000 | 0.213 | -0.286 | -0.041 | 0.038 | -0.009 |

N: number of individuals per lake; H_E_: expected heterozygosity; H_O_: observed heterozygosity; F­_IS_: inbreeding coefficient. Bolded H_O­_ values indicate significant departures from Hardy-Weinberg equilibrium (p<0.05).
